# Fish mouth deformity with fish eggs

**DOI:** 10.1016/j.igie.2023.01.007

**Published:** 2023-01-28

**Authors:** Andrew Canakis, Jennifer Grossman, Naomi Hardy, Peter Darwin

**Affiliations:** 1 Division of Gastroenterology & Hepatology, University of Maryland School of Medicine, Baltimore, Maryland, USA; 2 Department of Medicine, University of Maryland School of Medicine, Baltimore, Maryland, USA; 3 Department of Pathology, University of Maryland School of Medicine, Baltimore, Maryland, USA

An 81-year-old man whose identical twin was previously diagnosed with pancreatic cancer was referred for EUS after an unprovoked pulmonary embolism in which a CT incidentally revealed main pancreatic duct dilation. Upper endoscopy showed intraductal polypoid tissue at the papilla (fish egg appearance) with a fish mouth deformity extruding mucus (Fig. 1). Cold biopsy sampling of the pancreatic duct demonstrated papillary fragments of mucinous epithelium. On EUS the pancreatic duct was dilated to 9 mm with a mixed hypo- and isoechoic nodule confined to the duct (Fig. 2). These findings were consistent with a main duct intraductal papillary mucinous neoplasm. He subsequently underwent a pancreatoduodenectomy, where pathology confirmed noninvasive intraductal papillary mucinous neoplasm with focal extension into the ampulla and low-grade dysplasia (Fig. 3). He has been followed for 7 years with no further adverse events.

**Figure 1:**
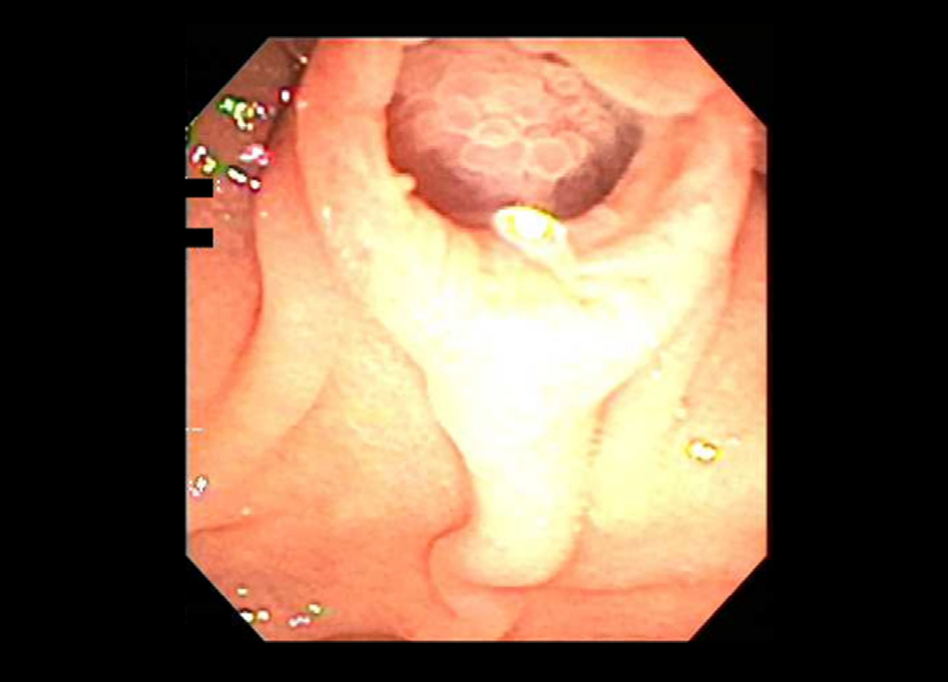
Upper endoscopy with a patulous papilla and fish mouth deformity extruding mucus.

**Figure 2:**
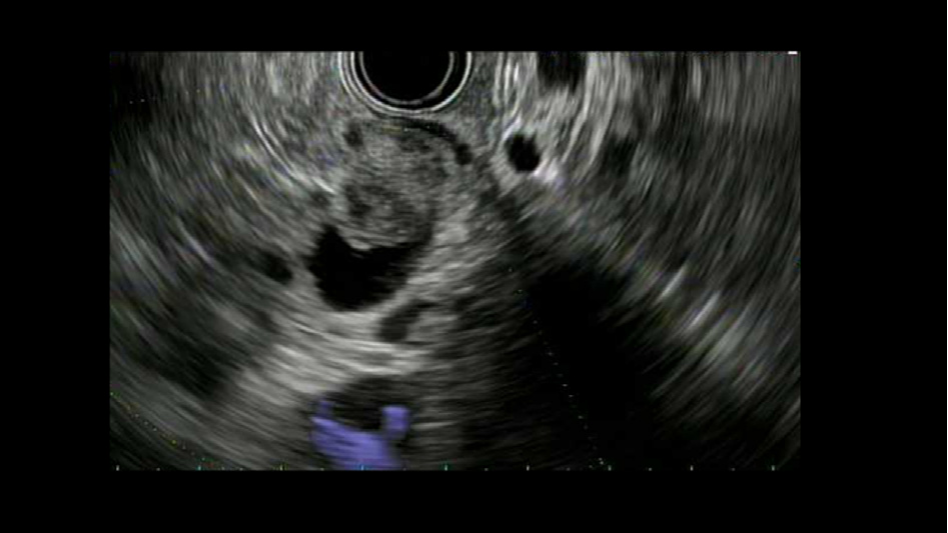
EUS demonstrating an intraductal mixed hypo- and isoechoic lesion within the lumen without invasion.

**Figure 3:**
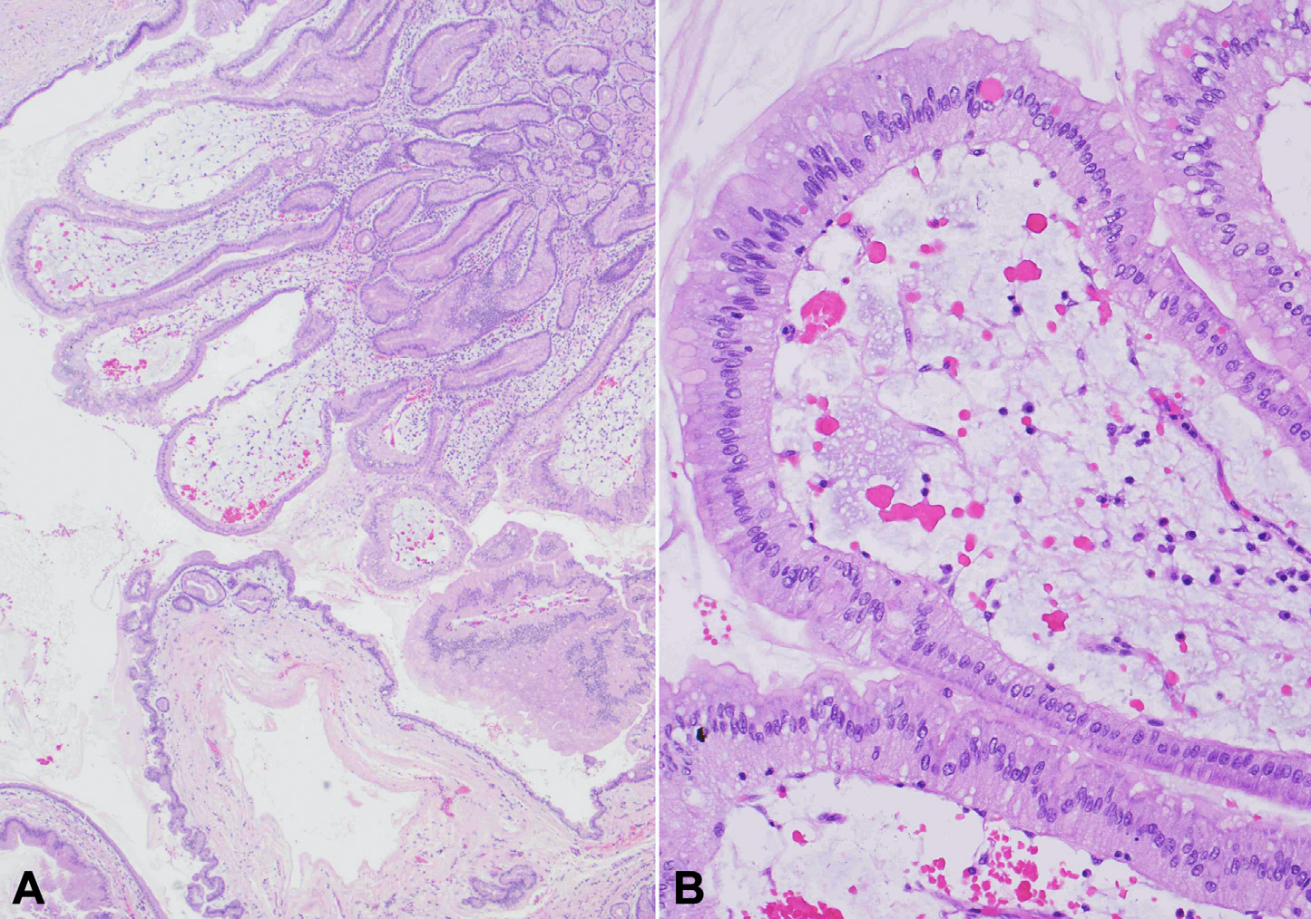
Whipple resection confirmed a noninvasive intraductal papillary mucinous neoplasm (**A,** H&E, 40×) with focal extension into the ampulla and low-grade dysplasia (**B,** H&E, 200×) with no evidence of high-grade dysplasia or invasion.

## Patient Consent

The authors have received appropriate patient consent for the publication of this article.

## Disclosure

*All authors disclosed no financial relationships.*

